# Large Cell Neuroendocrine Carcinoma of Gallbladder: A Case Report

**DOI:** 10.31729/jnma.8444

**Published:** 2024-02-29

**Authors:** Dhruba Narayan Sah, Oshan Shrestha

**Affiliations:** 1 Department of General Surgery, Nobel Medical College Teaching Hospital, Biratnagar, Morang, Nepal; 2 Department of Pathology, Nobel Medical College Teaching Hospital, Biratnagar, Morang, Nepal

**Keywords:** *carcinoma*, *case reports*, *cholecystectomy*, *gallbladder*

## Abstract

Large cell neuroendocrine carcinoma of the gallbladder is an extremely rare tumour with aggressive behaviour and a bad prognosis. Here, we report a case of a 65-year-old lady suspected of carcinoma of the gallbladder and underwent extended cholecystectomy. The histopathology report revealed neuroendocrine carcinoma of a large cell type of gall bladder infiltrating the liver and three periportal and pericholedochal lymph nodes. She had an uneventful perioperative period and was doing good till 6 months of follow-up. The only potentially curative treatment for large cell neuroendocrine carcinoma of the gallbladder is aggressive surgical resection, owing to its aggressive behaviour and bad prognosis.

## INTRODUCTION

Gallbladder neuroendocrine carcinoma (GB-NEC) is an aggressive malignancy with a bad prognosis. GB-NEC is extremely rare in clinical practice with few reports.^1^ Pure large cell neuroendocrine carcinoma (LCNEC) is even the rarest of rare entities.^2^ Aggressive surgical resection (and even resurgery) is the only potentially curative treatment. Vague symptoms of presentation and advanced stage of presentation account for the poorer prognosis of large cell neuroendocrine carcinomas of the gallbladder. The rarity of the occurrence and limited evidence on management make it difficult to formulate definitive guidelines for the management of LCNEC. We reported a case of gall bladder LC-NEC.

## CASE REPORT

**Figure 1:**
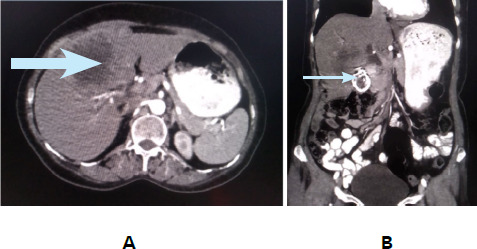
CECT images: (A) Axial view showing large heterogeneously enhancing hypodense lesion infiltrating in liver (B) Coronal view showing calcified gall bladder wall with few calculi within it.

A case of a 65-year-old lady without known comorbidities who had right upper abdominal pain for three weeks along with significant weight loss and anorexia without jaundice. Physical examination was unremarkable and routine investigations were within normal limits. Transabdominal ultrasonography revealed a heterogeneously hypoechoic vascular lesion in the gallbladder fossa with irregular anechoic areas with disruption of hepatic parenchyma and multiple calculi in the gall bladder largest 12 mm. Contrast-enhanced computerised tomography (CECT). It revealed a large heterogeneously hypodense lesion, engulfing a few calculi in the gallbladder fossa, infiltrating into the segments IVb and V of the liver showing patchy heterogeneous enhancement likely carcinoma of gallbladder infiltrating into segment liver without distant metastasis (Figure 1).

Liver function tests and tumour markers (CEA and CA-19-9) were normal.

**Figure 2:**
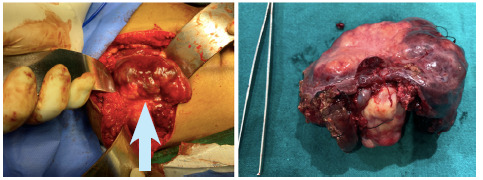
Intraoperative picture (up-arrow) showing hard gallbladder mass infiltrating to adjacent liver

A preoperative diagnosis of resectable gall bladder carcinoma was made. She underwent a staging laparoscopy followed by open extended cholecystectomy. Intraoperative findings were a gallbladder mass of 7 x 6 cm, hard infiltrating to adjacent liver segments IVb and V along with a few enlarged lymph nodes (Figure 2).

**Figure 3:**
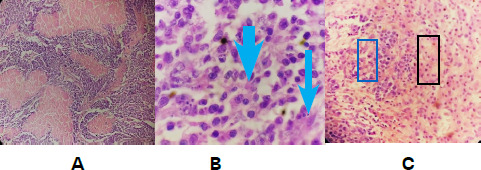
(A) Tumour cells in island and trabeculae with large areas of intervening geographic necrosis, (B) Tumor cells with moderate nuclear pleomorphism, coarse chromatin, and prominent nucleoli (arrow showing nucleoli), (C) Tumor cells in the left (rectangle/blue) infiltrating the hepatocytes in the right (rectangle/black).

The histopathology report revealed neuroendocrine carcinoma of large cell type (grade 3) of gall bladder infiltrating the liver and three periportal and peri choledochal lymph nodes were involved with the tumour (out of a total of six lymph nodes). The aortocaval lymph node was free from the tumour. The resection margins of the liver were not involved with the tumour (Figure 3).

She had an uneventful perioperative period and was discharged on the 9th postoperative day on a regular normal diet and oral analgesics. She was advised for chemotherapy but did not receive it. The patient was doing good till 6 months of follow-up.

## DISCUSSION

Early detection and resection are associated with better outcomes in neuroendocrine tumors (NET) of GB. GB-NEC represents 0.2% of all gastrointestinal NECs and 2.1% of all gall bladder carcinomas.^3^ As per various published literature, GB-NECs are diagnosed at advanced stages with distant metastasis; and are comparatively more aggressive as compared to conventional GB adenocarcinomas.^3-6^ Most of the reported cases were diagnosed postoperatively on histopathological evaluation of resected cholecystectomies or extended cholecystectomy specimens similar to this reported case.

The origin of GB-NEC is still under investigation. However, the most accepted one is chronic inflammation from cholelithiasis promoting metaplastic changes of gallbladder epithelial cells to neuroendocrine cells.^3^ The transformation from adenocarcinomas to NEC is another hypothesis.^6^ Neuroendocrine tumours of the digestive system have been classified based on mitotic figures and the Ki-67 index, regardless of the origin, size, or anatomic extent of the tumours.^7^ As per WHO 2019 updates, GB NEC encompasses small-cell and large-cell types of poorly differentiated NETs with mitotic rate >20mitoses/^2^mm^2^ and/or Ki-67 index >20%.^3^ The diagnosis of GB-NEC is based on typical morphological features and immunohistochemistry markers (chromogranin A, synaptophysin, and neuro-specific enolase); pathological results determine tumour grade and staging.^6^ We diagnosed the case based on the typical morphological features; immunohistochemical tests were not done due to unavailability at our setup and financial constraints.

GB-NEC is more prevalent in middle-aged and elderly females which is also consistent with this reported case of 65 year lady.^3^ They usually lack specific symptoms and typical imaging features and hence are difficult to diagnose preoperatively. Distant metastasis occurs even in the early stage though not detected in this case at the time of presentation. Additionally, there is no clear consensus on preoperative diagnosis, surgical strategies, and further management guidelines. In clinical practices the majority of GB-NEC have been dealt with as per GB adenocarcinomas and radical surgical resection is the optimal treatment. We also managed in line of carcinoma gallbladder with extended cholecystectomy.

This case had a survival of 6 months post-surgery. The prognosis of GB-NEC is very poor and the recurrence risk is very high.^5^ Very few pure LCNEC have been reported in the literature with a median survival of 8 to 10 months, so it becomes very difficult to propose the best management plan.^3,7^ Still, surgical resection improves overall survival and is the cornerstone in the management of GB-NEC.^5^ For neuroendocrine tumours of GB, cholecystectomy could be enough if detected in early stages (in situ and T1 tumours).^5,6^ Extended cholecystectomy (including GB, adjacent liver segments IVB & V along with adequate lymphadenectomy) should be the first line of treatment in patients with advanced GB-NEC without distant metastasis.^3,5^ Completion surgery is the standard practice for cases diagnosed post cholecystectomies.^7^ Due to its aggressive behaviour, repeated liver resection is also offered in patients with metastasis confined to the liver and with a good performance scale.^3,7^ Platinum-based postoperative adjuvant chemotherapy (cisplatin or carboplatin plus etoposide) is the main chemotherapeutic regimen to improve the prognosis for those in advanced stages with satisfactory results.^1,3^ Multimodality treatment with chemotherapy and radiotherapy is also advocated in patients with locally advanced or inoperable GB-NEC.^1,5^

Large cell neuroendocrine carcinoma of the gall bladder is one of the rarest of rare entities, with only a few cases reported. Even with tumour recurrence and poor overall survival, aggressive surgical management might be the best possible treatment strategy.
